# Enterococcus hirae Bacteremia Associated With Traumatic Soft Tissue Infection: A Case Report

**DOI:** 10.7759/cureus.74092

**Published:** 2024-11-20

**Authors:** Rita Jorge, Sara Teixeira, Marta Marques, José Pereira, José-Artur Paiva

**Affiliations:** 1 Department of Intensive Care Medicine, Hospital Beatriz Ângelo, Lisbon, PRT; 2 Department of Intensive Care Medicine, Centro Hospitalar Universitário de São João, Porto, PRT; 3 Department of Medicine, Faculty of Medicine, University of Porto, Porto, PRT

**Keywords:** case report, enterococcus bacteremia, major limb amputation, major trauma, soft tissue infection

## Abstract

In recent years, reports of *Enterococcus hirae* infections in humans have increased. Similarly to most known Enterococci, *E. hirae* has been identified mostly in bacteremia, urinary tract infections, infective endocarditis, and biliary tract infections. We present a case of *E. hirae* bacteriemia associated with traumatic soft tissue infection in a 77-year-old male patient, a polytrauma victim with a tibia-fibula open fracture after a forklift accident. Initially, the patient underwent a left below-the-knee amputation but it evolved poorly, with necrosis of the surgical stump. Debridement and antibiotics were started. Blood and soft tissue cultures identified *E. hirae*. An above-the-knee amputation was necessary, and the patient improved satisfactorily. Our case study helps to confirm the unexpected *E. hirae *in humans as well as report an unusual source of infection of this pathogen. Further studies and more case reports are needed to elucidate the clinical impact of *Enterococcus hirae *on humans.

## Introduction

*Enterococcus spp.* are gram-positive, facultatively anaerobic cocci and one of the usual residents of the gut microflora in humans and other mammals. Formerly known as group D streptococci, Enterococci are reported as the third most common cause of bacteremia and as an important cause of severe infections such as endocarditis, urinary tract infection and intra-abdominal abscesses [1]. Increased antibiotic use has led to their emergence as superinfecting nosocomial pathogens in humans [2].

While *E. faecalis* and *E. faecium* are this genus's most common human pathogens, *E. hirae* is a well-documented cause of infection in animals like birds, rats, chickens, or cattle [3,4]. However, reports of *E. hirae *infections in humans have increased in the last decades [4].

We present a case of *E. hirae* bacteriemia associated with traumatic soft tissue infection in a 77-year-old male patient with a tibia-fibula open fracture after a forklift accident.

## Case presentation

A 77-year-old male patient with arterial hypertension and benign prostate hyperplasia as past medical history was transferred to our department from a secondary hospital where he was admitted after a serious forklift accident. The patient was found under the forklift in his own garden with his pelvic region and inferior limbs incarcerated. He was locally assisted presenting with a Glasgow Coma Scale of 4 points, isocoric reactive pupils, hypotension and suspected head and pelvic trauma, alongside inferior limb trauma with open fractures and severe loss of substance. He was intubated, stabilized and transported to the nearest hospital. 

**Figure 1:**
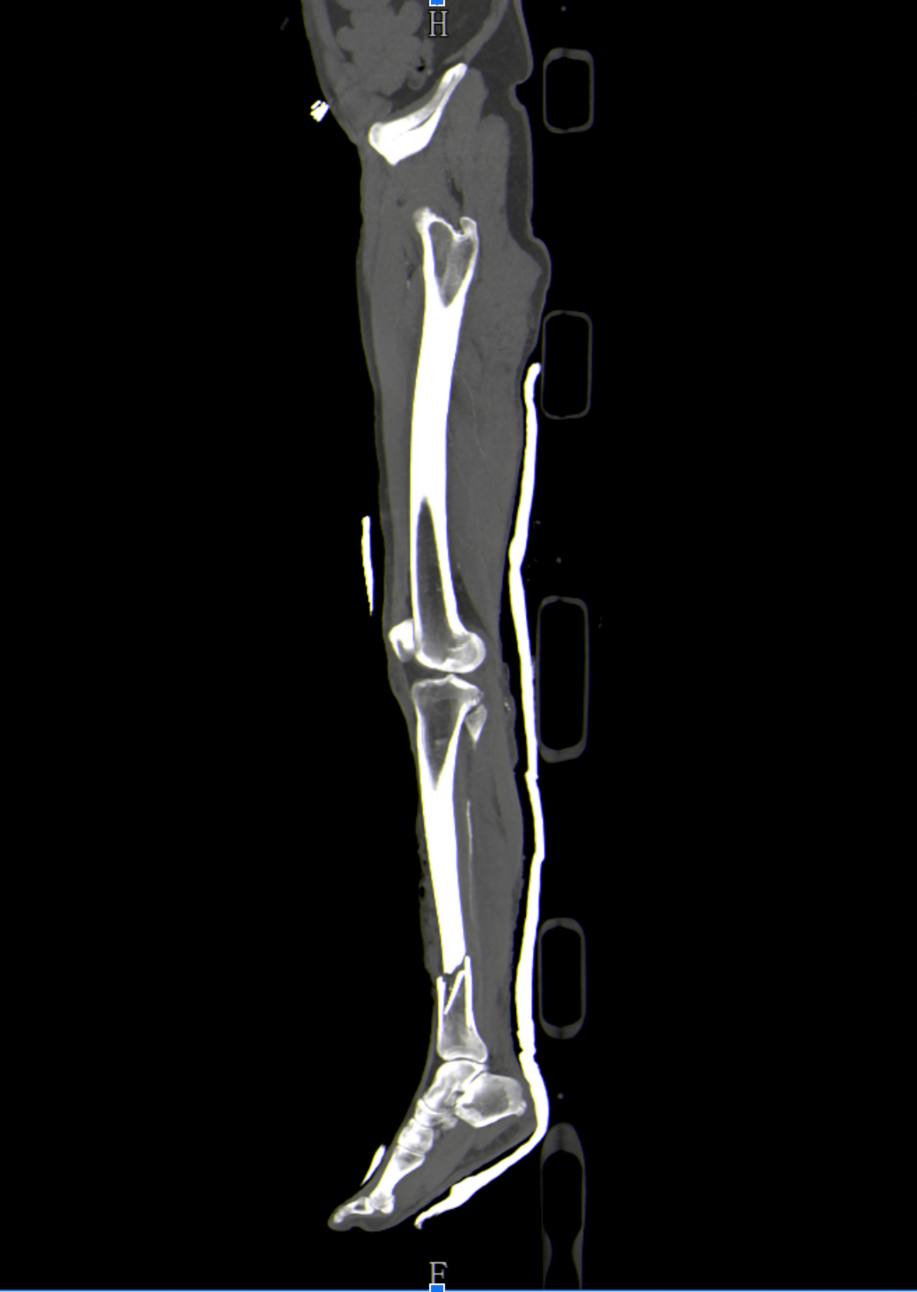
CT scan confirming left lower limb fracture. F: foot; H: head.

Extensive physical examination and CT scans confirmed multiple soiled wounds and degloving of the right inferior leg alongside unstable pelvic ring fractures (Tile C2) and left tibia-fibula open fracture (Gustilo and Anderson Grade III B) as shown in Figure 1.

The patient underwent surgical correction of severe soft tissue and skin injuries, fixation of pelvic fractures and left below-the-knee amputation with a Mangled Extremity Severity Score (MESS) score of 9. High doses of vasopressors and massive resuscitation with crystalloids and blood products were necessary. Prophylactic antibiotic therapy with cefazolin and gentamicin was established. The patient was then referred and transferred to our trauma ICU for continuation of care.

Besides other expected complications of polytrauma with brain injury, the left amputation stump and right leg soft tissue and skin injuries did not heal properly, developing extensive necrosis, fever and high inflammatory markers. Initially, the surgery team decided to do surgical debriding and negative pressure wound dressings. Blood and soft tissue cultures were collected and empiric piperacillin/tazobactam was initiated. Later, an above-the-knee amputation was necessary. Both initial blood and soft tissue (stump) cultures were positive for ampicillin-susceptible *E. hirae* without the recovery of other pathogens. Follow-up blood cultures were negative and endocarditis was excluded with a transthoracic echocardiogram. We maintained antibiotic therapy for 16 days and the patient underwent multiple surgeries and procedures throughout his hospital stay, including debriding, reconstruction, skin grafting and repair with satisfactory results and good recovery.

## Discussion

We report a case of severe infection due to *E. hirae* and emphasize an unusual source of infection for this pathogen, suggesting its possible wide-spectrum pathogenicity, especially in fragile individuals.

Originally described in 1985 by Farrow and Collins in young chickens [5], the first documented case of a human infection was reported by Gilad *et al*. in 1988, a case of a 49-year-old patient with end-stage renal disease affected by *E. hirae *septicemia [6]. Similar to his most known keens, it has been identified in bacteremia, urinary tract infections, infective endocarditis, biliary tract infections, pancreatitis and spondylodiscitis [4,7,8]. In fact, the relatively low level of antimicrobial resistance and low mortality suggest that *E. hirae* is more similar to *E. faecalis *than *E. faecium *[7].

Although *E. hirae *is a common pathogen in animals [9], human infection due to *E. hirae* remains rare, representing only 0.4% to 3.03% of Enterococci infections, according to the latest studies [8]. However, these numbers are possibly underestimated because of difficult isolation or inadequate identification until recently, overcome through the developments in matrix-assisted laser desorption ionization-time of flight mass spectrometry (MALDI-TOF MS) [4,10].

To our knowledge, this is the fourth case known of *E. hirae* isolated in wounds [4,11], the second one trauma-relatable. In Table 1, we present clinical and demographical characteristics of these patients. In most previously reported cases, a clear exposure before symptom onset it is not documented, independently of source infection [4,9,10]. In our case, a garden forklift accident with polytrauma and soiled wounds, it’s not possible to exclude it. In our critically ill patient, we preferred to maintain a wide-spectrum antibiotic despite the isolation of ampicillin-susceptible *E. hirae*, covering the high risk of multiple pathogens infection in an extensive soiled wound. There is no recommendation regarding the duration of antibiotic therapy in E. hirae infections. In our case, we decided to stop it five days after definitive adequate source control. 

**Table 1:** Enterococcus hirae reported wound cases.

Age gender/reference	Date	Underlying diseases	Immunosuppression	Diagnosis	Other organisms	Antibiotic therapy	Outcome
77 Male/This study	02/07/2023	Cardiovascular disease, benign prostate hyperplasia	None	Soft tissue necrosis trauma relatable	None	Piperacillin/Tazobactam	Alive
33 Male/ Bollam et al., 2016 [11]	21/10/2021	None	None	Acute osteomyelitis trauma relatable	Stenotrophomonas spp.	Vancomycin and Levofloxacin	Alive
82 Male/ Piccinini et al., 2023 [4]	23/09/2021	Cardiovascular, renal and lung disease, DM2, malignancy, skin disease	Malignancy; Steroids	Perianal abscess	Staphylococcus aureus	Piperacillin/Tazobactam	Alive
87 Male/ Piccinini et al., 2023 [4]	24/11/2016	Cardiovascular, renal and lung disease	None	Toe ulcer	None	Amoxicillin/Clavulanate	Alive

## Conclusions

Raising awareness of this rare clinical pathogen is essential to increase detection and timely treatment. Depending on the source of infection, prompt treatment includes source control and adequate use of antibiotics, guided by antimicrobial susceptibility testing whenever possible. Usually, the antimicrobial susceptibility of *E. hirae* is similar to that of *E. faecalis*, which is usually susceptible to penicillin. It is also important that medical teams are aware of the clinical significance of *E. hirae* as a new human infective pathogen. However, further studies and reports are needed to elucidate the true clinical epidemiology of *E. hirae* in humans. Since the prevalence of multidrug-resistant *Enterococcus spp.* worldwide has increased, it may be important to monitor the antimicrobial profile of *E. hirae*. 
